# Effects of Bitter Substances on GI Function, Energy Intake and Glycaemia-Do Preclinical Findings Translate to Outcomes in Humans?

**DOI:** 10.3390/nu13041317

**Published:** 2021-04-16

**Authors:** Peyman Rezaie, Vida Bitarafan, Michael Horowitz, Christine Feinle-Bisset

**Affiliations:** Adelaide Medical School and Centre of Research Excellence in Translating Nutritional Science to Good Health, Faculty of Health and Medical Sciences, University of Adelaide, Adelaide 5005, Australia; peyman.rezaie@adelaide.edu.au (P.R.); vida.bitarafan@adelaide.edu.au (V.B.); michael.horowitz@adelaide.edu.au (M.H.)

**Keywords:** bitter substances, gut hormones, gastric emptying, gastric motor function, food intake, postprandial blood glucose, preclinical studies, human studies, obesity, type 2 diabetes

## Abstract

Bitter substances are contained in many plants, are often toxic and can be present in spoiled food. Thus, the capacity to detect bitter taste has classically been viewed to have evolved primarily to signal the presence of toxins and thereby avoid their consumption. The recognition, based on preclinical studies (i.e., studies in cell cultures or experimental animals), that bitter substances may have potent effects to stimulate the secretion of gastrointestinal (GI) hormones and modulate gut motility, via activation of bitter taste receptors located in the GI tract, reduce food intake and lower postprandial blood glucose, has sparked considerable interest in their potential use in the management or prevention of obesity and/or type 2 diabetes. However, it remains to be established whether findings from preclinical studies can be translated to health outcomes, including weight loss and improved long-term glycaemic control. This review examines information relating to the effects of bitter substances on the secretion of key gut hormones, gastric motility, food intake and blood glucose in preclinical studies, as well as the evidence from clinical studies, as to whether findings from animal studies translate to humans. Finally, the evidence that bitter substances have the capacity to reduce body weight and/or improve glycaemic control in obesity and/or type 2 diabetes, and potentially represent a novel strategy for the management, or prevention, of obesity and type 2 diabetes, is explored.

## 1. Introduction

There has been increasing interest in the capacity of bitter substances to regulate energy intake and improve glycaemic control, based on reports from preclinical models (i.e., studies in cell cultures or animals) [1,2,3] that bitter substances have potent effects to secrete gastrointestinal (GI) hormones and slow gastric emptying. It is now well established that these gut functions play important roles in the regulation of both acute energy intake and postprandial glycaemia [4,5,6,7]. Thus, bitter substances may potentially represent a novel approach to the management or prevention of obesity and its comorbidities, particularly type 2 diabetes. This is an important issue, given that the efficacy of the majority of currently available treatments for obesity is limited. While lifestyle changes (reduction in energy intake, increased physical activity) lead to weight loss, which, even when modest, is associated with meaningful reductions in the risk of type 2 diabetes, long-term adherence to such interventions is usually poor [8]. The critical importance of the gut is attested to by the efficacy of bariatric surgery in producing sustained weight loss in the morbidly obese and marked improvement in glycaemic control in patients with type 2 diabetes, the latter even before major weight loss occurs [5]. Pharmacological options for the management of obesity are limited. Their use is often associated with adverse effects, particularly nausea, and their effects on body weight are usually limited, possibly because longer-term effective suppression of energy intake is dependent on the interaction of a number of mechanisms. The use of agonists of glucagon-like peptide-1 (GLP-1) in the management of type 2 diabetes and, more recently in higher dosage, obesity is now widespread [9,10,11,12]. Weight loss may be greater with higher-dose GLP-1 agonists, but as with all anti-obesity medication, the cost is substantial, particularly as sustained use may be required to prevent weight regain. Thus, there remains an urgent need to identify novel and inexpensive strategies that stimulate these gut functions without adverse effects, to promote the longer-term suppression of energy intake, clinically meaningful weight loss and, in type 2 diabetes, improved glycaemic control.

In studies on both cell lines and experimental animals bitter agonists have been shown to potently stimulate cholecystokinin (CCK), GLP-1 and ghrelin [3,13]. Furthermore, bitter tastants modulate contractility in mouse gastric muscle strips and slow gastric emptying [14,15]. These findings in preclinical studies have triggered considerable interest in the investigation of the effects of bitter agonists in clinical studies, to determine whether their effects can be reproduced in humans and, if so, if they are associated with reductions in energy intake and/or postprandial glycaemic excursions. As will be discussed, while some clinical studies have reported effects of bitter substances to stimulate GLP-1 and CCK [16,17], suppress ghrelin [14,18], modulate gastric motility [19] and/or suppress energy intake [16,18,19], the observed effects are inconsistent and often modest. Moreover, only two studies have, to date, reported effects to lower postprandial blood glucose in humans [17,20].

This review provides a brief summary of key aspects of the GI sensing of bitter substances by luminal bitter receptors. The focus is the evaluation of information relating to the effects of bitter substances on the secretion of gut hormones, gastric emptying and GI motility, energy intake and blood glucose in preclinical and clinical studies. Finally, we explore the question as to whether there is evidence to support the concept that bitter substances have the capacity to reduce body weight and/or improve glycaemic control in obesity and/or type 2 diabetes, with the inherent potential for their use in the prevention and/or management of these disorders.

## 2. Sensing of Bitter Substances in the GI Lumen

In contrast to sweet, umami or ‘fat’ tastes (which indicate nutrient availability), bitter taste is inherently aversive and has been viewed traditionally as having evolved primarily to warn against the presence of toxins, particularly in plants, or signal spoiled food [21]. Many foods and other substances taste bitter and are, therefore, unpleasant to ingest. However, bitter receptors, like receptors for nutrients [22,23], are present not only in the oral cavity [24], but throughout the GI tract on enteroendocrine cells [23,25,26]. The recognition that their GI sensing may trigger beneficial metabolic effects, has, in recent years, fuelled substantial interest in a better understanding of GI bitter sensing and the investigation of the GI effects of bitter tastants. Unlike dietary macronutrients, bitter substances are devoid of energy and, accordingly, do not contribute to overall caloric intake, which represents an inherent advantage.

### 2.1. Sources of Bitter Compounds

Natural bitter-tasting compounds are contained in many foods that provide nutrition and contribute to health, including extracts of many plants (e.g., *Hoodia gordonii*, *Gentiana scabra*, *Humulus lupulus* L. flower, bark of the cinchona tree) as well as plant-based foods (e.g., *Brassica* vegetables and certain fruit), and processed dairy products, and include phenols, flavonoids and glucosinolates, amongst many others. They can also be found in animal-derived foods or generated during the process of food aging or spoilage [27,28]. Furthermore, Maillard and fermentation reactions can generate bitter compounds. Many chemically synthesised compounds, including denatonium benzoate, phenylthiocarbamide or 6-n-propylthiouracil (the latter two are often used to determine bitter taste sensitivity experimentally), as well as many drugs, have a strong bitter taste [29,30,31,32,33]. Bitter compounds are not only numerous (the number has been estimated to be in the tens of thousands [34]), but are also each characterised by a unique and diverse structure, consisting of phenols, esters, fatty acids, hydroxy fatty acids, amines, flavonoids, amongst many others, indicative of a broad range of bitter chemotypes [31,34].

### 2.2. Bitter Taste Receptors

Bitter tastants are detected by taste 2 receptors (TAS2Rs), which are members of the GPCR superfamily of receptors [34,35]. A large number of receptor subtypes has been identified in various species, including current totals of 25 in humans and >30 in rodents [36]. While some bitter compounds activate a single TAS2R subtype, the majority activates a range of TAS2R subtypes, although the combination of subtypes varies [34]. For example, salicin, from willow bark, activates TAS2R16, and both phenylthiocarbamide and propylthiouracil interact only with TAS2R38 [34]. In contrast, quinine, an extract from the bark of the cinchona tree, activates nine (TAS2R4, 7, 10, 14, 39, 40, 43, 44, 46) and denatonium benzoate eight (TAS2R4, 8, 10, 13, 39, 43, 46, 47) subtypes, of which five are in common with quinine. Quassin, an extract of the tropical quassia tree, activates five subtypes (TAS2R4, 10, 14, 46, 47) [34], of which four are in common with both quinine and denatonium benzoate. Bitter substances that activate differing combinations of receptor subtypes include sodium cyclamate, which activates TAS2R1 and 38, and sinigrin, found in cruciferous plants, which activates TAS2R16 and 38 [34]. That bitter substances activate different combinations of receptor subtypes (with varying overlap between individual compounds) may account for why only a limited number of TAS2Rs have the capacity to detect so many bitter compounds. In the absence of a comprehensive understanding of either the function(s) of each receptor subtype or the location and distribution of receptor subtypes on specific cells (e.g., enteroendocrine cells), the variability in receptor activation across bitter substances represents a major challenge to the clarification of their physiological roles and therapeutic potential.

Single nucleotide polymorphisms have also been described in TAS2Rs and shown to be associated with individual differences in bitter taste perception, food preferences and/or food consumption [32,33,37,38]. Thus, while bitter taste perception is reproducible in a given individual, the effects of bitter substances also vary between individuals. A well-documented example of a polymorphism is the ability to taste phenylthiocarbamide and 6-n-propylthiouracil, which is genetically determined by the TAS2R38 gene [39]. Based on the molecular structure of this receptor, individuals can be categorised into two common phenotypes, i.e., those that can taste these compounds and those that are ‘non-tasters’ [38,39]. The effect(s) of gene polymorphisms on the sensitivity to bitter compounds in extra-oral locations, including the GI tract, and how genetic variations may modify these effects remain to be clarified.

In contrast to knowledge relating to the characteristics of nutrient receptors, including their localisation and distribution along the GI tract, which has been reviewed in detail elsewhere [22,40,41,42,43,44,45,46,47,48], information regarding the regional distribution of TAS2Rs, and the functions of specific subtypes, is limited. Evaluation of the putative TAS2Rs 1 to 12 gene transcripts (except 11, which is not a functional gene) from rat antral, fundic and duodenal mucosa demonstrated a greater number of bitter receptor subtypes in the duodenum than the stomach [25]. In another study, which investigated locations of TAS2Rs by RNA sequencing on intestinal cells of rhesus macaques, only TAS2R1, 3, 4, 5, 19, 20, 38 and 46 were expressed in the duodenum, ileum and colon, with greater expression of TAS2R38 in the small, than the large, intestine [49]. Based on these observations, targeted administration of bitter compounds directly into the small intestine may potentially be associated with greater potency. However, an important caveat is that animal receptor subtypes may not correspond to those in humans. Knowledge relating to the functions of individual receptor subtypes is limited, but recent studies have defined roles for specific receptor subtypes in the regulation of gut hormones. For example, TAS2R5 and 38 may be involved in the release of GLP-1 from human L-cells and HuTu-80 cells, respectively [50,51], and TAS2R5 and 6 in ghrelin secretion [52]. An improved understanding of the distribution of TAS2Rs along the GI tract in humans, and the specific functions of individual receptor subtypes, will be critical to effective targeting of the administration of bitter substances to optimise bitter agonist-gut interactions.

## 3. Effects of Bitter Substances on Gut Hormone Release

A large number of gut hormones have been identified and many, including CCK, PYY, GLP-1, glucose-dependent insulinotropic polypeptide (GIP), ghrelin, motilin, oxyntomodulin, are pivotal to the regulation of gut motor function, energy intake and/or blood glucose [53,54,55,56,57,58]. CCK, PYY, GLP-1 and ghrelin are probably the best characterised. CCK, PYY and GLP-1 all have potent effects to modulate gastropyloroduodenal motility and slow gastric emptying, and reduce energy intake [5]. The critical involvement of endogenous hormones in these effects was confirmed by studies, in which administration of specific hormone receptor antagonists was shown to attenuate the suppression of energy intake [59,60,61]. These hormones also have potent effects to reduce energy intake when administered intravenously [62,63,64]. In contrast to CCK, PYY and GLP-1, ghrelin, whose circulating concentrations are high in the fasting state and thought to play a role in the initiation of eating, is suppressed by nutrients [57]. Once released, gut hormones exert their effects in part by activating specific receptors on vagal afferents [65,66], but may also have direct effects in brain centres involved in appetite regulation [5]. GLP-1, which is one of the two ‘incretin’ hormones (the other being GIP), is a physiological modulator of postprandial glycaemia, stimulating insulin and suppressing glucagon in a glucose-dependent manner [67], and slowing gastric emptying [11,53,68,69]. GLP-1 agonists and dipeptidyl peptidase-4 (DPP-4) inhibitors, which prevent degradation of endogenously secreted active GLP-1, are now used extensively in the management of type 2 diabetes to improve blood glucose control [10,12].

The presence of bitter substances in the GI lumen, following oral consumption or direct luminal administration, initiates a cascade of intracellular events culminating in the release of a number of gut hormones [3,70,71]. A substantial number of studies have evaluated the effects of bitter substances on gut hormone secretion in preclinical studies (Table 1). A range of bitter substances appear to have potent stimulatory effects, particularly on CCK and GLP-1, as well as ghrelin, in the models used. In contrast, effects on PYY are poorly defined. Only a small number of studies has been performed in humans [16,17,18,19,20,72,73,74,75,76] (Table 2). These studies have yielded inconsistent outcomes and, perhaps surprisingly in view of the preclinical outcomes, if positive, the observed effects have been modest. The following sections will review evidence on the effects of bitter substances on the secretion of CCK, GLP-1, PYY and ghrelin, based on studies in both preclinical models and humans.

### 3.1. Cholecystokinin

#### 3.1.1. Outcomes of Preclinical Studies

The effects of bitter substances on CCK secretion have only been investigated in animal or human cell lines, and gut tissues ex vivo [2,13,77,78,79]. In mouse STC-1 or Caco-2 cells, matured hop bitter acids (MHBA, an oxidised bitter extract from the hops flower, *Humulus lupulus* L.), denatonium benzoate and phenylthiocarbamide all stimulated CCK release dose-dependently, MHBA 20- to 80-fold and denatonium benzoate 100- to 300- fold, while phenylthiocarbamide only resulted in a 1.5-fold increase [2,77,78]. In both excised rat intestinal tissue and the human enteroendocrine cell line, HuTu-80, steroid glycosides (extracted from the succulent plant, Hoodia gordonii), an agonist for hTAS2R7 and 14, stimulated CCK 1.5- to 3-fold, respectively, effects abolished by administration of the TAS2R14 antagonist, compound 03A3 [13]. Moreover, 1,10-phenanthroline, which selectively activates TAS2R5, stimulated CCK, from rat duodenal segments; in contrast, the TAS2R14-specific agonist, flufenamic acid, apparently decreased CCK release [74].

**Table 1:** Effects of bitter substances on gut hormone secretion in preclinical models.

Bitter Tastant	Model	Doses Given/Location of Delivery	Approx. Equivalent Dose in a 70-kg Human ^1^	Observed Effect	Ref #
Berberine	STC-1 cells	1, 10, 100, 200 µM	-	↑ GLP-1	[80]
	NCI-H716 cells	1, 10, 100, 200 µM	-	↑ GLP-1	[81]
Chloroquine	Human fundic cells	0.3–10 mM	-	↑ Ghrelin	[52]
Denatonium benzoate	STC-1 cells	1–10 mM	-	↑ CCK	[2]
	NCI-H716 cells	2, 10 mM	-	↑ GLP-1, PYY	[3]
	Human fundic mucosa	0.5, 1, 5 mM	-	↑ Ghrelin	[52]
	Mice	1 mg/kg/oral	≈70 mg	↑ GLP-1	[3]
	Mice	60 μmol/kg/IG	≈1.8 g	↑ GLP-1	[1]
Epicatechin gallate	MGN3-1 cells	10 μM	-	↓ Ghrelin	[82]
		500 μM	-	↑ Ghrelin	
Erythromycin A	Human fundic mucosa	0.03, 0.3, 1 mmol/L	-	↑ Ghrelin	[52]
Flufenamic acid	Rat ex-vivo segments:				[79]
	- duodenal	10 µM	-	↓ CCK	
	- ileal	10 µM	-	↑ GLP-1 ↔ PYY	
Gallic acid	MGN3-1 cells	10 μM	-	↓ Ghrelin	[82]
Gentiana scabra extract	NCI-H716 cells	100–750 μg/mL	-	↑ GLP-1	[83]
Hoodia gordonii	HuTu-80 cells	10 mM	-	↑ CCK	[13]
KDT501 ^2^	STC-1 cells	10 μM	-	↑ GLP-1	[84]
	Mice	150 mg/kg/oral	≈10 g	↑ GLP-1	[84]
Mature hop bitter acids	STC-1 cells	50, 100, 200 μg/mL	-	↑ CCK, GLP-1 ↔ PYY	[78]
Ofloxacin	NCI-H716 cells	10, 50, 100 mM	-	↑ GLP-1	[85]
1,10-Phenanthroline	NCI-H716 cells	10–500 µM	-	↑ GLP-1	[51]
	Human fundic mucosa	0.1, 1 mM	-	↑ Ghrelin	[52]
	Rat ex-vivo segments:				[79]
	- duodenal	150 µM	-	↑ CCK	
	- ileal	150 µM	-	↑ GLP-1 ↔ PYY	
Phenylthiocarbamide	STC-1 cells	2, 5, 10 mM	-	↑ CCK	[2]
	Caco-2 cells	10 mM	-	↑ CCK	[77]
	Human fundic cells	0.3–10 mM	-	↑ Ghrelin	[52]
Propylthiouracil	Human fundic cells	0.3–10 mM	-	↑ Ghrelin	[52]
	Mice	200 mg/kg/IG	≈14 g	↑ GLP-1	[50]
Qing-Hua granules	Mice	3.75, 7.5, 15 g/kg/d/IG	≈263–1050 g	↑ GLP-1	[86]
Quinine hydrochloride	NCI-H716 cells	0.5, 1, 2 mM	-	↑ GLP-1	[3]
	Mice	160 μmol/kg/IG	≈4 g	↔ GLP-1, ghrelin	[1]
Vanillic acid	Rat ileal segments	151.17 µM	-	↑ GLP-1	[79]
Wild bitter gourd	STC-1 cells	100, 500, 1000 µg/mL	-	↑ GLP-1	[87]
	Mice	5 g/kg/IG	≈350 g	↑ GLP-1	[87]

CCK, cholecystokinin; GLP-1, glucagon-like peptide-1; IG, intragastric; PYY, peptide YY. ^1^ Only calculated for whole-animal studies, ^2^ bitter compound derived from isohumulone, an extract from the hops plant.

#### 3.1.2. Outcomes of Studies in Healthy Humans

In healthy males, administration of amarasate^TM^ (a supercritical CO_2_ extract from New Zealand native hops), in a dose of 500 mg, given either in an acid-resistant capsule (to target small intestinal bitter receptors) or a standard capsule (to release its content in the stomach) was reported to stimulate CCK in response to a subsequent lunch, consumed 60 min and 30 min after intestinal- and gastric-targetted administration, respectively, as well as a snack, consumed 120 min after lunch [72], although the magnitude of the effect was not reported. In contrast, in healthy males and females, 18 mg quinine hydrochloride, ingested orally in an acid-resistant capsule did not increase absolute plasma CCK concentrations, although the change in plasma CCK relative to baseline was slightly greater 30 min after consumption of an ad-libitum standardised buffet meal (~0.9 ± 0.6 vs. 0.5 ± 0.8 ng/mL) [16]. Moreover, 60-min intraduodenal infusions of quinine hydrochloride, providing 75 mg [74], or 37.5 mg, 75 mg and 225 mg [76], had no effect on plasma CCK in healthy, lean men. In these latter studies the relatively low infusion rate may have been insufficient to reach a critical threshold for activation of TAS2Rs [20].

**Table 2:** Effects of bitter substances on gut hormone secretion in healthy humans.

Bitter tastant	Model	Doses Given/Location of Delivery	Observed Effect	Ref #
Amarasate^TM 1^	Males	500 mg in acid-resistant or standard capsules/oral	↑ CCK, GLP-1, PYY	[72]
Denatonium benzoate	Females	1 μmol/kg bolus/IG [≈32 mg] ^2^	↔ Ghrelin	[19]
Quinine hydrochloride	Males	10 µmol/kg bolus/IG [≈270 mg]	↓ Ghrelin	[18]
	Males and females	18 mg in acid-resistant capsule/oral	↑ CCK	[16]
	Males and females	75 mg/ID over 60 min	↔ CCK, GLP-1, PYY	[74]
	Females	10 μmol/kg bolus/IG [≈270 mg]	↓ Ghrelin	[75]
	Males	37.5, 75, 225 mg/ID over 60 min	↔ CCK	[76]
	Males	275, 600 mg bolus/IG 30 min before meal	↑ GLP-1	[17]
	Males	600 mg bolus/IG 60 min before meal, ID 30 min before meal	↑ GLP-1	[20]
Secoiridoids ^3^	Males and females	100 mg/oral (microencapsulated) incorporated in custard	↑ GLP-1 ↔ PYY, ghrelin	[73]

CCK, cholecystokinin; GLP-1, glucagon-like peptide-1; ID, intraduodenal; IG, intragastric; PYY, peptide YY. ^1^ Supercritical CO_2_ extract from New Zealand native hops, ^2^ approximately equivalent dose in a 70-kg human, ^3^ bitter compound derived from *Gentiana lutea* plant.

### 3.2. Glucagon-Like Peptide-1

#### 3.2.1. Outcomes of Preclinical Studies

The effects of bitter compounds on GLP-1 secretion have been studied extensively [1,3,50,51,78,79,80,81,83,84,85,86,87]. A number of bitter compounds have been shown to stimulate GLP-1 in both cell line and animal studies. For example, berberine, found in several bitter plants, stimulated GLP-1 in human enteroendocrine NCI-H716 cells, and phenylthiourea in HuTu-80 cells around 1.5- to 2-fold, by activating TAS2R38 [50,80,81]. The latter effect was diminished, but not abolished, by silencing the TAS2R38 using non-coding small interfering RNA [50], indicating that while this receptor is involved in phenylthiourea-induced GLP-1 release, other mechanisms also contribute. In NCI-H716 cells, 1,10-phenanthroline stimulated GLP-1 via activation of TAS2R5, and denatonium benzoate via a range of TAS2R subtypes, including TAS2R4, 43, and 46 [3,51]. In mouse SCT-1 cells, application of the bitter compounds, MHBA and KDT501, pure derivatives of isohumulone, extracted from hops, stimulated GLP-1 release 1.5- to 2.5-fold [78,84], and the effect of KDT501 was attenuated by silencing the TAS2R108 with small hairpin RNA, implicating a role for this receptor [84]. Moreover, 1,10-phenanthroline, as well as the selective TAS2R14 agonists, vanillic acid and flufenamic acid, stimulated GLP-1 release from ileal segments from rat ~1.5-fold [79].

In mice, oral administration of 1 mg/kg denatonium benzoate and 5 g/kg wild bitter gourd, prior to glucose gavage, or 200 mg/kg propylthiouracil, stimulated GLP-1 secretion ~2–3 fold in all studies [3,50,87]. These doses were equivalent to ~70 mg, ~350 g and ~14 g in a 70 kg person, and were, accordingly, high. In diet-induced obese mice, acute oral gavage of KDT501, in the dose of 150 mg/kg, prior to an oral glucose load (1 g/kg), stimulated GLP-1 levels 3-fold within 15 min, while chronic treatment (150 mg/kg daily) for 17 weeks resulted in a more than 10-fold increase in plasma GLP-1 within four days, with the effect sustained over the treatment period [84]. Moreover, in obese mice, intragastric administration of denatonium benzoate (60 μmol/kg), but not quinine hydrochloride (160 μmol/kg), for four weeks also stimulated plasma GLP-1 ~1.5-fold [1].

#### 3.2.2. Outcomes of Studies in Healthy Humans

**Figure 1:**
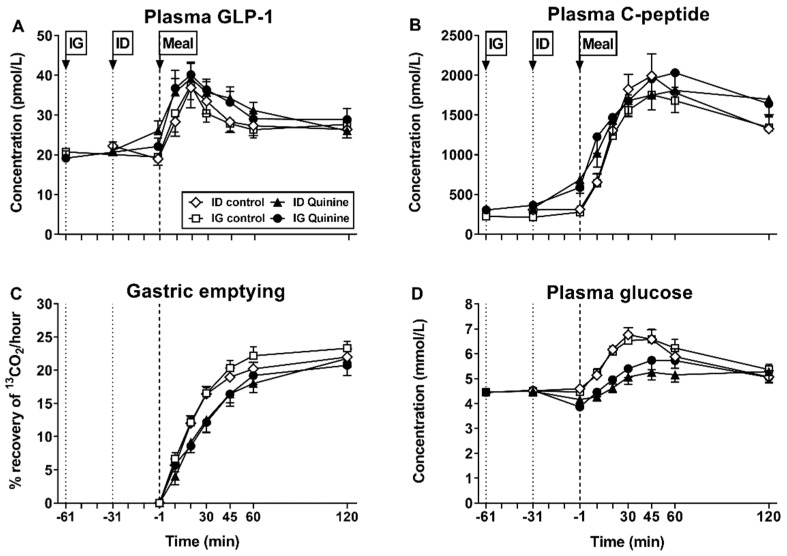
Effects of quinine on (**A**) plasma glucagon-like peptide-1 (GLP-1), (**B**) plasma C-peptide, (**C**) gastric emptying (measured using a ^13^C-acetate breath test) and (**D**) plasma glucose in 14 healthy men. Quinine, given as quinine hydrochloride in a dose of 600 mg, or control, was administered either intragastrically (IG, at t = −61 min), or intraduodenally (ID, at t = −31 min), before a mixed-nutrient drink (500 kcal, 74 g carbohydrates), consumed at t = −1 min. IG and ID administration of quinine comparably (**A**) increased plasma GLP-1 concentration before, and in response to, the drink, (**B**) increased plasma C-peptide, before, and during the first 10 min in response to, the drink, (**C**) slowed gastric emptying of the drink, and (**D**) reduced plasma glucose before, and particularly following, the drink (Adapted from ref. [20]).

Amarasate^TM^, in a dose of 500 mg, also stimulated GLP-1, however, no information was provided about the magnitude of the effect [72]. Two studies that evaluated the effects of quinine (given as quinine hydrochloride) provided evidence that the timing of administration influenced the effect on GLP-1 [17,20]. In the first study, intragastric administration of quinine, in doses of 275 mg and 600 mg, 30 min before a 350-mL mixed-nutrient drink (500 kcal, 74 g carbohydrates) did not stimulate plasma GLP-1 during the first 30 min (i.e., in response to quinine alone), but increased GLP-1 modestly (by ~15 pmol/L) following the drink [17]. In the second study [20], 600 mg quinine was administered either intragastrically 60 min before, or intraduodenally 30 min before, a nutrient drink, and in both conditions plasma GLP-1 was increased modestly, by quinine alone, and further following the drink (Figure 1). These observations suggest that intragastric administration may require a longer time to achieve a comparable effect to that of intraduodenal administration, implying that exposure of small intestinal bitter receptors to quinine may be necessary for stimulation of GLP-1. In contrast to these observations, continuous intraduodenal infusion of quinine, in a dose of 75 mg, over 60 min had no effect on GLP-1 [74], perhaps because a threshold concentration was not achieved at the location of the receptors. The 600-mg dose of quinine hydrochloride in the above studies [17,20] is comparable to the acute therapeutic dose of quinine (500 mg) used for malaria treatment.

### 3.3. Peptide YY

#### 3.3.1. Outcomes of Preclinical Studies

Information relating to the effects of bitter compounds on PYY secretion is both limited and inconsistent [3,78,79]. While, in murine NCI-H716 cells, denatonium benzoate (2 mmol/L) stimulated PYY release ~2.3 fold [3], in murine STC-1 cells, administration of MHBA was reported to have no effect [78]. Since PYY is co-localised with GLP-1 in enteroendocrine L-cells [88], the effects of bitter substances on PYY would be expected to be comparable to those on GLP-1. It is, accordingly, surprising that this has not been investigated, particularly, given the important role of PYY in the regulation of energy intake [54].

#### 3.3.2. Outcomes of Studies in Healthy Humans

While amarasate^TM^ was reported to stimulate plasma PYY [72], small intestinal administration of 100 mg bitter secoiridoids [73] and a 60-min intraduodenal infusion of quinine, in the dose of 75 mg [74], were found to be ineffective.

### 3.4. Ghrelin

#### 3.4.1. Outcomes of Preclinical Studies

The effects of bitter compounds on ghrelin secretion have been investigated in a number of studies in both cell and rodent models; the majority of these have been performed by one research group [1,14,52,82]. Denatonium benzoate (0.5, 1 and 5 mmol/L), chloroquine (0.5, 1 and 5 mmol/L), 1,10-phenanthroline (0.1, 1 mmol/L), a selective agonist for TAS2R5, and erythromycin A (0.03, 0.3, 1 mmol/L), a TAS2R10-specific agonist, all stimulate ghrelin secretion in cultures of human fundic mucosa ~2-fold [52]. In a study, which evaluated the effects of phenolic extracts from grape seed, effects varied substantially between compounds, depending on the study conditions [82]. For example, epicatechin gallate, an agonist of mTAS2R14 and mTAS2R39, inhibited ghrelin secretion in a mouse gastric ghrelinoma cell line, MGN3-1, at the low dose of 10 μM by ~20%, while stimulating ghrelin 2-fold at a high dose (500 μM) [82]. Gallic acid also inhibited ghrelin release from MGN3-1 cells by ~20% (although only a 10 μM dose was tested), and by ~33% in rats pre-treated with gallic acid for 8 days [82]. In wild-type mice, intragastric administration of a mixture of bitter compounds containing denatonium benzoate, quinine hydrochloride, phenylthiocarbamide, propylthiouracil and D-salicin, stimulated plasma ghrelin ~2-fold [14]. In contrast, in obese mice on a high-fat diet, daily gavage with quinine (160 μmol/kg) had no effect on plasma ghrelin [1]. Thus, a range of bitter compounds stimulate ghrelin secretion in-vitro, while the outcomes of animal studies are inconsistent.

#### 3.4.2. Outcomes of Studies in Healthy Humans

Intragastric administration of quinine, in a dose of 10 μmol/kg, suppressed plasma ghrelin in males [18] and females [75] modestly, and intragastric administration of denatonium benzoate, in a dose of 1 μmol/kg in healthy females [19], or administration of 100 mg bitter secoiridoids into the small intestine in healthy males and females [73], had no effect. Thus, evidence for a ghrelin-suppressant effect of bitter substances is limited.

Taken together, there is persuasive evidence that bitter substances have the capacity to stimulate gut hormones via a number of specific bitter receptor subtypes, and that subtypes vary between compounds and hormones, however, much more work is required to characterise their involvement. Identification of the specific role of specific receptor subtypes will facilitate targeted use of bitter compounds for defined outcomes. The doses used, particularly in the animal models have, in many cases, been very high, as assessed by calculating equivalent doses in humans. Thus, whether such doses could be used safely in humans, and/or whether lower doses have substantial effects, remains to be determined. The possibility that, because of the high doses used, some of the observed effects may reflect ‘non-specific’ effects of bitter compounds, requires clarification. For example, some bitter agonists used in these studies (e.g., phenylthiocarbamide), at the doses administered, have, in other studies, been shown to induce potent Ca^2+^ responses in control cells devoid of bitter receptors [89]. It should also be appreciated that because no studies have included positive controls, e.g., dietary nutrients, interpretation of the relative magnitude, and the relevance, of the observed effects on hormone release is confounded. Finally, there is a lack of information about the tolerability of bitter compounds, and the possibility that, at least some of, the observed effects reflect an aversive response, requires investigation.

In contrast to preclinical studies, the effects of bitter substances on gut hormone secretion in humans are largely inconclusive, moreover, the range of bitter compounds studied is limited. While it appears that, in line with preclinical studies, certain bitter compounds are associated with stimulation of GLP-1 in humans, the particular receptor subtype(s) involved remain to be identified. For example, whether stimulation of TAS2R38, which has been identified as a potent mediator of GLP-1 in in-vitro studies using human tissue [50], plays a role or other receptor subtypes (e.g., TAS2R5 or 14) are involved, given that quinine does not stimulate TAS2R38. Studies are required to systematically evaluate the effects of specifically selected compounds (e.g., those activating only specific single receptor subtypes) including targeting specific GI regions (e.g., the duodenum). Given that frequently much higher doses were used in preclinical studies, it will also be important to determine whether pharmacological doses are, indeed, necessary to achieve substantial effects and, if so, whether these occur in the absence of adverse effects.

## 4. Effects of Bitter Substances on Gastric Emptying and Gastrointestinal Motility

Gastric emptying regulates the transfer of chyme, and, therefore, the rate of nutrient entry, to the small intestine. Slowing of gastric emptying reflects closely coordinated changes in the motor function of the stomach and small intestine, which include relaxation of the proximal stomach, tonic and phasic pyloric contractions and suppression of antral and duodenal pressures [90,91]. As gastric emptying progresses, food components, particularly dietary nutrients, interact with specialised receptors located on the surface of enteroendocrine cells, triggering the secretion of gut hormones [22,40,42,43,44,48], which, at least in part, mediate nutrient-induced slowing of gastric emptying. The latter prolongs gastric distension and, thereby, enhances the feeling of fullness after a meal [92,93]. While both proximal and distal gastric filling contribute to the perception of fullness [93,94,95], antral content has been shown to be related closely to energy intake [95], and is, therefore, likely to be a major ‘intragastric’ mechanism.

Gastric emptying also plays a key role in the postprandial glycaemic response; in this context, regulation of the small intestinal delivery, and subsequent absorption, of glucose, as well as the release of glucoregulatory hormones, including GLP-1 and GIP, are of particular relevance. Thus, gastric emptying accounts for ~35% of the variance in the early (approximately first 30–45 min) rise in postprandial glucose in healthy individuals and those with diabetes [96,97]. It is now also recognised that the primary action of GLP-1 to lower blood glucose probably occurs via slowing of gastric emptying [98,99].

### 4.1. Outcomes of Preclinical Studies

Preclinical studies suggest that bitter compounds slow gastric emptying and modulate gastric motility [14,15,100,101] (Table 3). For example, in normal-weight mice, intragastric administration of phenylthiocarbamide (30 µmol/kg) or denatonium benzoate (60 µmol/kg) slowed gastric emptying [15], while in another study intragastric administration of denatonium benzoate in the higher dose of 10 mM had no effect [14]. The reason(s) for the discrepancy between the two studies are not clear. In the former study [15], the effect of denatonium benzoate, but not phenylthiocarbamide, was abolished by probenecid (50 mg/kg), an inhibitor of the TAS2R16, 38 and 43 subtypes, suggesting that the effect of denatonium benzoate may be mediated via TAS2R43 (denatonium benzoate does not activate TAS2R16 and 38), while raising the possibility that the effect of phenylthiocarbamide, which only activates TAS2R38, on gastric emptying does not involve bitter receptor activation. The mixture of denatonium benzoate, quinine hydrochloride, phenylthiocarbamide, propylthiouracil and D-salicin, described above, also slowed gastric emptying, which was unaffected by co-administration of the CCK antagonist, devazepide, or the GLP-1 antagonist, exendin (9–39) [14], indicating that slowing of gastric emptying induced by this mixture, and in the doses administered, was not mediated by CCK or GLP-1, but, as evidenced by the inhibition of the electrical field stimulation-induced activity in both antral and duodenal smooth-muscle strips, was likely to reflect a direct inhibitory effect on gastric smooth muscle cells [14,15]. In contrast, in mice, oral administration of swertiamarin, an extract from the *Swertia japonica* plant, in doses of 250 or 500 mg/kg, was reported to accelerate gastric emptying [100].

A few studies have evaluated the effect of bitter compounds on gastric contractile activity, and the reported effects vary substantially among the various compounds, as well as across species. For example, in mouse fundic and antral smooth-muscle strips, denatonium benzoate triggered concentration-dependent tonic fundic contraction (maximal at 100 μM) and antral phasic activity, but at higher concentrations (1 mM) induced fundic relaxation and inhibited antral activity [15]. In contrast, phenylthiocarbamide only induced dose-related relaxation of fundic muscle, while completely inhibiting activity in antral muscle, and salicin was ineffective [15]. These fundus-relaxing and antrum-inhibitory effects would be consistent with the observed slowing of gastric emptying in the in-vivo investigations [15]. In contrast to the effects observed in mouse tissue [15], in guinea pigs, oral (0.2 nmol/mL) and intragastric (0.1 and 1 nmol/kg) administration of denatonium benzoate increased gastric accommodation, consistent with gastric relaxation, while the higher dose of 30 µmol/kg inhibited accommodation [101]. Finally, in mice, oral administration of swertiamarin, in doses of 250 or 500 mg/kg, increased small intestinal motility [100]. The observed differences between some of the bitter compounds (and between species) are likely to be attributable to a number of factors, including the differential involvement of specific bitter taste receptor subtypes and variations in the sensitivity to different compounds.

There is, accordingly, evidence that bitter substances slow gastric emptying and modulate aspects of GI motility, although how some of the observed effects on motility (e.g., increased fundic tone) can be reconciled with the slowing of gastric emptying observed in vivo remains to be determined.

**Table 3:** Effects of bitter substances on gastric emptying and gastric motor function in preclinical models.

Bitter Tastants	Model	Doses Given/Location of Delivery	Approx. Equivalent Dose in a 70-kg Human ^1^	Observed Effect	Ref #
Chloroquine	Mouse fundic and antral smooth-muscle strips	10–100 μM >1 mM	− −	↑ Phasic antral activity ↔ Tonic fundic contraction ↓ Phasic antral activity ↑ Fundic relaxation	[15]
Denatonium benzoate	Mouse fundic and antral smooth-muscle strips	10–100 μM >1 mM	− −	↑ Tonic fundic contraction and phasic antral activity ↑ Fundic relaxation ↓ Phasic antral activity	[15]
	Mice	60 µmol/kg/IG	≈1.8 g	↓ Gastric emptying ↓ Fundic and antral motility	[15]
	Mice	10 mM/IG	≈0.04 g	↔ Gastric emptying	[14]
	Rats	10 mM/IG	≈0.04 g	↓ Gastric emptying	[102]
	Guinea pigs	0.2 nmol/mL/oral 0.1, 1 nmol/kg/IG 30 µmol/kg/IG	≈0.003 mg ≈0.003–0.03 mg ≈0.98 g	↑ Gastric accommodation ↓ Gastric accommodation	[101]
Phenylthiocarbamide	Mouse fundic and antral smooth-muscle strips	10 μM–10 mM	−	↑ Fundic relaxation ↓ Antral activity	[15]
	Mice	30 µmol/kg/IG	≈3.2 g	↓ Gastric emptying ↓ Fundic and antral motility	[15]
Salicin	Mouse fundic and antral smooth-muscle strips	10 μM–10 mM	−	↔ Fundic and antral contractility	[15]
Swertiamarin	Mice	250, 500 mg/kg/oral	≈17.5 and 35 g	↑ Gastric emptying ↑ Small intestinal motility	[100]
Mixture of DB, PTC, PTU, quinine HCl, D-salicin	Mice	DB 10 mM; PTC 10 mM; PTU 5 mM; quinine 1.5 mM; D-salicin 5 mM/IG	DB ≈ 46 mg; PTC ≈ 15 mg; PTU ≈8 mg; quinine ≈ 5 mg; D-salicin ≈ 15 mg	↓ Gastric emptying	[14]

DB, denatonium benzoate; HCl, hydrochloride; IG, intragastric; PTC, phenylthiocarbamide; PTU, propylthiouracil. ^1^ Only calculated for whole-animal studies.

### 4.2. Outcomes of Studies in Healthy Humans

A number of studies have evaluated the effect of bitter compounds on gastric emptying [16,17,19,20,103,104], with the majority reporting no effects [16,17,19,103] (Table 4). For example, in healthy males and females, 18 mg quinine hydrochloride, ingested orally in an acid-resistant capsule (to target release in the duodenum) did not affect gastric emptying of a 480-kcal solid meal [16]. Moreover, in healthy females, intragastric administration of 1 μmol/kg denatonium benzoate had no effect on gastric emptying of a 500-kcal pancake [19]. Finally, in healthy males, intragastric administration of quinine hydrochloride, in doses of 275 and 600 mg, had no effect on gastric emptying of a mixed-nutrient drink (350 mL; 500 kcal) consumed 30 min later [17]. In contrast, when the higher dose of quinine (600 mg) was administered either intragastrically 60 min, or intraduodenally 30 min, before the nutrient-drink, gastric emptying was slowed, with no difference between the two routes of administration (Figure 1) [20], providing evidence that intestinal exposure to bitter substances may be critical.

Information relating to the effect of bitter substances on gastroduodenal motility is also limited and inconsistent. In healthy males and females, intragastric administration of denatonium benzoate attenuated fundic relaxation after a nutrient drink [15], while, in healthy females, intragastric administration of quinine hydrochloride was reported to reduce ‘fluctuations’ in fasting antral motility, without affecting duodenal motility [75], and, in healthy males, a 60-min intraduodenal infusion of quinine, delivering overall doses of 37.5 mg, 75 mg or 225 mg, did not affect antral, pyloric or duodenal pressures [76].

Thus, while animal studies demonstrate an effect of bitter substances to slow gastric emptying, the effects on gastric emptying in humans, as well as any contributing contractile mechanisms, remain uncertain. Both the timing and location of delivery may be important.

## 5. Effects of Bitter Substances on Energy Intake

Based on the findings in preclinical studies of potent modulation by bitter substances of key GI factors, i.e., gut hormones and gastric emptying, involved in the acute regulation of food intake, there has been considerable interest in evaluating the effects of bitter substances on eating. This section will discuss studies that have investigated effects on caloric intake.

### 5.1. Outcomes of Preclinical Studies

A number of preclinical studies have evaluated the effects of bitter compounds on food intake [1,14,79,105,106,107,108,109] (Table 5). For example, in rats, oral gavage of an extract from *Hoodia gordonii* (in doses of 6.25–50 mg/kg) for three days suppressed intake from an ad libitum standard laboratory diet for eight days post-administration, and reduced body weight [107]. In rats with impaired glucose tolerance, oral administration of berberin (doses: 93.75, 187.5 or 562.5 mg/kg) for eight weeks reduced food intake from an ad libitum high-fat laboratory chow and also attenuated weight gain [108]. Moreover, in rats, ad-libitum consumption of a powdered chow diet containing quinine sulphate (0.75% by weight) for 32 days was associated with a reduction in food intake and body weight during the first two days of treatment, although food intake returned to control levels within 2 weeks [109]. While these observations suggest that these bitter compounds have an intake-suppressant effect, the bitter taste associated with oral consumption may have been offensive and discouraged food intake. However, the fact that bitter substances also reduce food intake when administered directly into the stomach argues against this possibility. For example, in mice, acute intragastric administration of a mixture of bitter compounds, including denatonium benzoate, quinine hydrochloride, phenylthiocarbamide, propylthiouracil and D-salicin, while increasing food intake during the first 30 min post-administration, suppressed intake during the subsequent 4 h [14]. The initial increase may potentially reflect stimulation of ghrelin, while the subsequent inhibition was interpreted as reflecting slowing of gastric emptying (despite continued elevation of ghrelin) [14].

Moreover, in diet-induced obese mice, intragastric administration of 60 µmol/kg denatonium benzoate for 4 weeks reduced food intake from a liquid meal [1], an effect associated with GLP-1 stimulation. Interestingly, intragastric administration of quinine, in a dose of 160 μmol/kg and using the same study protocol [1], failed to either stimulate GLP-1 or reduce food intake. Furthermore, despite apparent discrepant effects on food intake, both denatonium benzoate and quinine reduced weight gain in mice [1], suggesting that the effect of quinine on body weight may be mediated by other mechanisms. In support of this concept, some bitter substances, including bitter orange extracts [110] and a mixture of salicin and naringin [111], have been reported to increase resting energy expenditure and diet-induced thermogenesis in the absence of a reduction in food intake, associated with weight loss. Finally in rats, intragastric administration of agonists of hTAS2R5 (e.g., 1,10-phenantroline in a dose of 200 mg/kg) and hTAS2R14 (e.g., vanillic acid in a dose of 252 mg/kg), but not hTAS2R39 agonists, reduced food intake from an ad-libitum standard chow diet 3, 12 and 20 h post-administration [74]. The food intake-suppressant effect of 1,10-phenantroline was associated with CCK and GLP-1 stimulation, and the effect of vanillic acid with GLP-1 stimulation, as measured using excised intestinal tissue [74].

### 5.2. Outcomes of Studies in Healthy Humans

There is little information about the effects of bitter compounds on appetite or energy intake in humans, and the reported outcomes are inconclusive [16,17,18,19,72,73,74,76] (Table 6). For example, in healthy females, while intragastric administration of denatonium benzoate (1 µmol/kg) increased ‘satiety’ and reduced hunger after a standardised meal, there was no significant suppression of energy intake from a subsequent ad-libitum buffet meal [19]. In contrast, in healthy males and females, ingestion of 18 mg quinine hydrochloride in an acid-resistant capsule modestly reduced energy intake from an ad-libitum meal 60 min later by ~82 kcal [16], and an intragastric bolus of 10 μmol/kg (~250 mg) quinine hydrochloride, in healthy females, modestly reduced energy intake from a highly palatable chocolate milk shake, by ~67 kcal [18]. The latter effect was associated with ghrelin suppression and an increased activity of brain centres involved in the control of feeding, including the hypothalamus and hedonic regions [18]. On the other hand, intraduodenal infusion of 75 mg quinine hydrochloride, in healthy volunteers (6 men, 9 women) [74], or doses of 37.5, 75 and 225 mg over 60 min [76], or intragastric bolus administration, in doses 275 and 600 mg [17], in healthy males, did not affect energy intake from ad libitum meals. Only two studies have reported more substantial reductions in energy intake. In one study, including healthy males and females, consumption of a microencapsulated extract of bitter compounds, derived from Gentiana lutea root, incorporated in a standardised custard breakfast (314 kcal), decreased total energy intake on that day by a substantial 22% (~340 kcal) [73]. In the second study in healthy males, administration of amarasate^TM^, in a dose of 500 mg, reduced energy intake from an ad-libitum lunch by 277 kcal, and from a snack, provided 2 h after lunch, by 225 kcal [72].

Taken together, preclinical studies indicate consistent effects of bitter substances to suppress food intake, associated with a reduction in body weight. In contrast, information about the effects of bitter substances on energy intake in healthy humans remains limited, and findings are inconsistent, although there is evidence that some bitter compounds appear to have a potent suppressive effect.

**Table 6:** Effects of bitter substances on energy intake in healthy humans.

Bitter Tastants	Model	Doses Given/Location of Delivery	Type of Meal or Diet	Observed Effects	Ref #
Amarasate^TM 1^	Males	500 mg in acid-resistant or standard capsules/oral	Ad libitum lunch and snack	↓ Energy intake	[72]
Denatonium benzoate	Females	1 µmol/kg bolus/IG (≈30 mg) ^2^	Ad libitum meal (2330 kcal, 291 g CHO, 94 g F, 55 g Prot)	Trend for ↓ energy intake	[19]
Quinine hydrochloride	Males and Females	18 mg in acid-resistant capsule/oral	Ad-libitum meal (50% CHO, 31% F, 19% Prot)	↓ Energy intake	[16]
	Males and Females	75 mg/ID over 60 min	Ad libitum meal (160 kcal/100 g; 7.1 g Prot, 11 g CHO, 9.4 g F)	↔ Energy intake	[74]
	Females	10 μmol/kg bolus/IG (≈250 mg)	Ad libitum palatable chocolate milkshake	↓ Energy intake	[18]
	Males	37.5, 75, 225 mg/ID over 60 min	Ad libitum meal (2300 kcal, 52% CHO, 27% F, 21% Prot)	↔ Energy intake	[76]
	Males	275, 600 mg bolus/IG 30 min before meal	Ad libitum meal (2300 kcal, 52% CHO, 27% F, 21% Prot)	↔ Energy intake	[17]
Secoiridoids ^3^	Males and females	100 mg/oral (micro-encapsulated) incorporated in custard	Ad libitum meal (3 h later)	↔ Energy intake	[73]

CHO, carbohydrate; F, fat; ID, intraduodenal; IG, intragastric; Prot, protein. ^1^ Supercritical CO_2_ extract of New Zealand native hops plant, ^2^ approx. equivalent dose in a 70-kg human, ^3^ bitter compound derived from the *Gentiana lutea* plant.

## 6. Effects of Bitter Substances on Postprandial Blood Glucose

The potent effects of bitter substances to stimulate glucoregulatory hormones, particularly GLP-1, and slow gastric emptying (a major determinant of postprandial blood glucose), have provided a rationale for the investigation of the capacity of these compounds to reduce postprandial blood glucose levels. The latter is of major clinical relevance, since it is now appreciated that in type 2 diabetes postprandial glycaemic excursions are the major, and in many cases dominant, determinant of average glycaemic control, as assessed by measurement of glycated haemoglobin (HbA1c).

### 6.1. Outcomes of Preclinical Studies

A consistent effect of bitter compounds to lower postprandial blood glucose has been reported [3,83,87,110] (Table 7). For example, in db/db mice, oral administration of denatonium benzoate, in a dose of 1 mg/kg, reduced blood glucose levels by ~2 mg/dL, 20–40 min after glucose gavage (5 g/kg) [3]. Similarly, in db/db mice, oral administration of an extract from the root of the Gentia scabra plant immediately before an oral glucose load (5 g/kg), reduced blood glucose—the dose of 100 mg/kg by ~1 mg/dL within 90 min, and the 300 mg/kg dose by ~2 mg/dL within 40 min [83]. Moreover, in mice, a high-fat diet containing 5% extract of bitter gourd for 5 weeks reduced blood glucose AUC during a 90-min oral glucose tolerance test (2 g/kg) by ~350 mg/dL∙min [87]. The glucose-lowering effects of denatonium benzoate, and *Gentia scabra* root and bitter gourd extracts, were associated with stimulation of GLP-1 and insulin [3,83,87]. Moreover, the glucose-lowering effect of bitter gourd extract was attenuated by co-administration of the GLP-1 receptor antagonist, exendin (9–39)amide [87], supporting a role for GLP-1 in glucose-lowering.

The effect of bitter compounds to improve glycaemia may also relate to effects on insulin sensitivity [110]. For example, in diabetic KK-A^y^ mice, the substantial postprandial glucose-lowering effect of a diet containing 0.18% of either isohumulone or isocohumulone (two major bitter acids derived from hops), fed for 14 days, was associated with activation of peroxisome proliferator activated receptors, PPARα and PPRAγ, intranuclear transcription factors known to improve insulin resistance [110]. Moreover, in C57BL/6N high-fat diet-fed mice, administration of isocohumulone (in doses of 10 and 100 mg/kg) for 14 days, reduced the plasma glucose response to an oral glucose load (1 g/kg body weight), associated with an improvement in insulin sensitivity [110].

**Table 7:** Effects of bitter substances on postprandial blood/plasma glucose in preclinical models and healthy humans.

Bitter Tastants	Model	Doses Given/Location of Delivery	Approx. Equivalent Dose in a 70-kg Human	Type of Meal	Observed Effects	Ref #
**(A) Preclinical models**						
Denatonium benzoate	Mice	1 mg/kg/oral	≈70 mg	OGTT (5 g glucose/kg BW)	↓ Blood glucose	[3]
Gentiana scabra extract	Mice	100, 300 mg/kg/oral	≈7–21 g	OGTT (5 g glucose/kg BW)	↓ Blood glucose	[83]
Isocohumulone ^1^	Mice	10, 100 mg/kg/oral	≈0.7–7 g	OGTT (1 g glucose/kg BW)	↓ Plasma glucose	[110]
Wild bitter gourd	Mice	High-fat diet containing 5% extract/oral	-	OGTT (2 g glucose/kg BW)	↓ Blood glucose	[87]
(**B) Healthy humans**						
Quinine hydrochloride	Males	37.5, 75, 225 mg/ID over 60 min		N/A ^2^	↔ Blood glucose (fasting)	[76]
	Males	275, 600 mg/IG 30 min before meal		Mixed-nutrient drink (500 kcal, 74 g CHO)	↓ Plasma glucose	[17]
	Males	600 mg/IG 60 min before meal, ID 30 min before meal		Mixed-nutrient drink (500 kcal, 74 g CHO)	↓ Plasma glucose	[20]
Secoiridoids ^3^	Males and females	100 mg/oral (micro-encapsulated) incorporated in custard		Custard + biscuits (314 kcal, 45.1 g CHO)	↔ Blood glucose	[73]

BW, body weight; CHO, carbohydrate; ID, intraduodenal; IG, intragastric; N/A, not applicable; OGTT, oral glucose tolerance test. ^1^ Bitter acid derived from hops plant, ^2^ blood glucose was measured in the fasting state, ^3^ bitter compound derived from the *Gentiana lutea* plant.

Accordingly, a number of bitter substances have potent effects to lower postprandial blood glucose, although evidence to support the involvement of specific receptor subtypes or the role of hormones, particularly GLP-1, is limited. No studies have hitherto evaluated the relationship between the effects on blood glucose with slowing of gastric emptying.

### 6.2. Outcomes of Studies in Healthy Humans

The effects of bitter substances on postprandial blood glucose have been evaluated in three studies in humans [17,20,73] (Table 7). Intragastric administration of quinine, in doses of 275 and 600 mg, reduced the glucose response to a mixed-nutrient drink containing 74 g carbohydrate in healthy males, associated with enhanced stimulation of GLP-1 and insulin after the drink, but without any effect on gastric emptying [17]. In a subsequent study, 600 mg quinine given either intragastrically 60 min, or intraduodenally 30 min, before a nutrient-drink also reduced blood glucose substantially, with no difference between the two routes of administration (Figure 1) [20]. Moreover, quinine stimulated both plasma GLP-1 and C-peptide (a measure of insulin secretion) immediately before the meal, and also slowed gastric emptying. In this study, the early postprandial plasma glucose response was shown to be related directly to gastric emptying, and inversely to plasma C-peptide immediately before the drink [20], suggesting that both slowing of gastric emptying, as well as insulin stimulation, contributed to glucose lowering. The role of GLP-1 in the observed glucose-lowering is uncertain, given that GLP-1-induced insulin secretion is glucose-dependent requiring plasma concentrations to be elevated above ~7 mmol/L [111]. Thus, it is possible that the glucose-lowering effect of quinine reflects a direct action of quinine on pancreatic beta cells to stimulate insulin [112]. In contrast, the lack of effect of a 60-min intraduodenal in-fusion of quinine, delivering overall doses of 37.5, 75 and 225 mg, in the absence of a carbohydrate source [76], is not surprising. Finally, in healthy males and females, consumption of 100 mg microencapsulated of *Gentiana lutea* plant extract in a custard and biscuit breakfast did not affect blood glucose over the subsequent 180 min [73]. However, only one dose was studied.

Taken together, the available studies, although limited, suggest a substantial effect of quinine, when administered into the upper GI lumen, to lower postprandial blood glucose.

## 7. Is There Evidence That Bitter Substances Reduce Body Weight and Improve Blood Glucose Control in Obesity and Type 2 Diabetes?

While observations from preclinical studies have provided evidence for potent effects of bitter substances to reduce food intake and lower postprandial blood glucose, in part related to gut hormone secretion, leading to weight loss, observations in healthy humans are less clear-cut, and, for the main part, effects, if any, were modest. Despite the latter, conclusions are frequently drawn as to the major implications of these findings for the development of novel management strategies for obesity and type 2 diabetes. We now address whether this concept is supported adequately by studies in people with obesity and/or type 2 diabetes.

A number of studies have reported associations between oral bitterness perception and body mass index (BMI) [113,114,115,116,117]. For example, higher body weights have been reported to be associated with reduced bitter perception in both adults (i.e., BMI > 28 kg/m^2^ vs. BMI < 28 kg/m^2^) [116] and children and adolescents (i.e., body weight > 97th percentile vs. body weight < 90th percentile) [117]. While impaired perception of sweet taste has been reported in people with type 2 diabetes [118,119], findings relating to bitter taste perception are less consistent. Some [119,120], but not all [121], studies have reported reduced oral bitter perception in type 2 diabetes. For example, only ~57% of males and females with well-controlled type 2 diabetes, compared with 72% of healthy individuals, were able to identify a bitter stimulus correctly in a taste identification task, independent of gender, disease duration or chronic glycaemic control, in one study [120], while another study found no difference [121], although perception of other tastes, including sweet, sour and salty was reduced.

GI morphological alterations, e.g., reduced numbers of enteroendocrine cells, both in the stomach and duodenum, have been reported in morbidly obese, compared with lean, people [122], which may potentially affect bitter sensing in the GI lumen, with consequences for gut hormone release, food intake and/or blood glucose control. There is evidence that the GI expression of some bitter taste receptor subtypes may be altered in obesity [52,123]. For example, expression of TAS2R38 has been reported to be augmented in colonic enteroendocrine cells producing CCK, GLP-1 and PYY in overweight and obese people [123], although the effect on hormone release by bitter substances was not evaluated. Moreover, in cultures of human gut mucosa, expression of TAS2R10 in ghrelin cells, which was found in both gastric and jejunal tissue, was greater in obese individuals in the stomach, but not the jejunum [52]. This was associated with differential effects on ghrelin stimulation by denatonium benzoate; thus, while denatonium benzoate stimulated ghrelin release from the fundus in both lean and obese, the effect was reduced in the obese, and in the jejunum, ghrelin stimulation only occurred in lean individuals [52], suggesting that sensitivity of ghrelin-secretory cells to this bitter agonist may be altered.

There is limited information regarding the effects of bitter sensing on glucose homeostasis [85,124]. While associations have been found between genetic variants of subtypes, including TAS2R9 and TAS2R38, with the glucose response to an oral glucose tolerance test [124] or GLP-1 and insulin release [85], the effects of bitter substances on blood glucose control have, to date, not been investigated in clinical studies.

No studies have investigated the effects of bitter substances on gut hormone release and gastric emptying in obesity and type 2 diabetes, and whether such effects, if any, are associated with a reduction in energy intake, weight loss and long-term improvement in blood glucose control.

## 8. Summary and Future Directions

**Figure 2:**
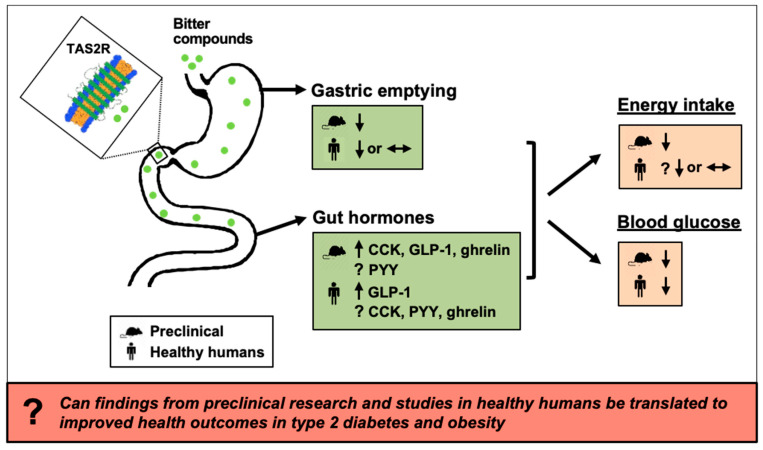
Schematic summarising current knowledge of effects of bitter substances on the secretion of gut hormones, including cholecystokinin (CCK), glucagon-like peptide-1 (GLP-1), peptide YY (PYY) and ghrelin, gastric emptying, energy intake and blood glucose, based on the outcomes of both preclinical studies (cell and animal models) and studies in healthy humans. TAS2R, bitter taste receptor.

In preclinical models bitter substances unequivocally have potent effects on upper GI functions, particularly the secretion of gut hormones, including CCK, GLP-1 and ghrelin, associated with reductions in food intake and body weight, and a reduction in postprandial blood glucose excursions, including in models of obesity and type 2 diabetes. In contrast, the limited clinical studies have yielded much more inconsistent outcomes and only been performed in healthy humans. These studies are indicative of an effect of bitter compounds to stimulate GLP-1 and to lower postprandial glucose, and to reduce energy intake modestly (Figure 2).

There are a number of important issues that need to be addressed. The range of available bitter substances, activating a large variety of combinations of bitter receptor subtypes, not all of which may be relevant to outcomes, represents a major challenge to the systematic evaluation and comprehensive understanding of their effects. At least some of the inconsistencies in findings between humans and preclinical studies may be because the latter have often applied high doses of bitter compounds. Thus, evaluation of a wide range of bitter compounds, including a broad range of doses is required to determine efficacy, as well as tolerability. Moreover, no studies have directly compared the effects of different bitter compounds, administered intestinally, when adjusted for their bitterness, i.e., at identical intensities, to assess their effects independent of bitterness intensity. There is evidence that rats cannot discriminate orally between equibitter solutions of quinine and denatonium benzoate [125]. Characterisation of the specific bitter receptor subtypes involved in the regulation of GI functions, particularly the release of gut hormones, as well as their regional distribution along the human GI tract, may be pivotal to targeted use of specific bitter agonists to achieve defined outcomes, e.g., the release of specific gut hormones and/or slowing of gastric emptying, as well as potency of these effects. The roles of gut hormones and gastric emptying in the effects of bitter substances on energy intake and blood glucose require additional investigation to define whether these are causal or coincidental. Moreover, clarification of the role of genetic variations of receptor subtypes in interindividual differences in bitterness perception, and their relationships with effects on GI functions, energy intake and blood glucose control, is desirable.

A major omission is the absence of studies relating to the effects of bitter substances in people with obesity and/or type 2 diabetes. Such studies, which would initially evaluate acute effects, represent a priority. If the outcomes are positive, longer-term trials to establish whether acute effects translate to sustained weight loss and long-term improvements in glycaemic control will be warranted. Only then will it be known whether the encouraging findings from laboratory-based studies can be translated into innovative strategies for the management, treatment or prevention of obesity and type 2 diabetes.

## Figures and Tables

**Table 4:** Effects of bitter substances on gastric emptying and gastroduodenal motor function in healthy humans.

Bitter Tastants	Model	Doses Given/Location of Delivery	Observed Effect	Ref #
Denatonium benzoate	Females	1 μmol/kg bolus/IG (≈30 mg) ^1^	↔ Gastric emptying	[19]
	Males and females	1 µmol/kg bolus/IG	↓ Fundic relaxation	[15]
Naringin	Males and females	1 mM bolus (≈580 mg)/IG	↔ Gastric emptying	[103]
Quinine hydrochloride	Males and females	18 mg in acid-resistant capsule/oral	↔ Gastric emptying	[16]
	Females	10 μmol/kg bolus/IG [≈270 mg]	↓ ‘Fluctuations’ in antral motility ↔ Duodenal motility	[75]
	Males and females	0.198 mM [≈72 mg]/IG	↔ Gastric emptying	[103]
	Males	37.5, 75, 225 mg/ID over 60 min	↔ Antropyloroduodenal motility	[76]
	Males	275, 600 mg bolus/IG 30 min before meal	↔ Gastric emptying	[17]
	Males	600 mg bolus/IG 60 min before meal, ID 30 min before meal	↓ Gastric emptying	[20]
Quinine sulphate	Females	10 mg bolus/oral	↓ Gastric emptying	[104]

ID, intraduodenal; IG, intragastric. ^1^ Approximately equivalent dose in a 70-kg human.

**Table 5:** Effects of bitter substances on food intake and body weight in preclinical models.

Bitter Tastants	Model	Doses Given/Location of Delivery	Approx. Equivalent Dose in a 70-kg Human	Type of Meal or Diet	Observed Effects	Ref #
Berberine	Rats	93.75, 187.5, 562.5 mg/kg/oral	≈6.5, 13, 39 g	Ad libitum high-fat chow	↓ Food intake ↓ Weight gain	[108]
Denatonium benzoate	Mice	60 μmol/kg/IG	≈1.8 g	Mixed-nutrient liquid meal	↓ Food intake ↓ Weight gain	[1]
Epicatechin	Rats	300 mg/kg/IG	≈21 g	Ad libitum standard chow diet	↓ Food intake	[79]
Hoodia gordonii extract	Rats	6.25–50 mg/kg/oral	≈0.4–3.5 g	Ad libitum standard diet (55% CHO, 15% Prot, 3% F)	↓ Food intake ↓ Body weight	[107]
*Humulus lupulus* L. extract	Mice	2–5% of diet/oral	-	Ad libitum standard (77% CHO, 9.7% F, 13.9% Prot) or high-fat diet (546 kcal/100 g)	↓ Food intake ↔ Weight gain	[105]
	Rodents	0.2–1.2% of diet/oral	-	Ad libitum standard diet (77% CHO, 9.7% F, 13.9% Prot) or high-fat diet (60% F, 14% CHO, 26% Prot)	↓ Food intake ↓ Weight gain	[106]
1,10-Phenanthroline	Rats	200 mg/kg/IG	≈14 g	Ad libitum standard chow diet	↓ Food intake	[79]
Quinine hydrochloride	Mice	160 μmol/kg/IG	≈4 g	Mixed-nutrient liquid meal	↔ Food intake ↓ Weight gain	[1]
Quinine sulphate	Rats	0.75% of diet/oral	-	Ad libitum powdered chow diet	↓ Food intake ↓ Body weight	[109]
Vanillic acid	Rats	252 mg/kg/IG	≈17 g	Ad libitum standard chow diet	↓ Food intake	[79]
Mixture of DB, PTC, PTU, quinine HCl, D-salicin	Mice	DB 10 mM; PTC 10 mM; PTU 5 mM; quinine 1.5 mM; D-salicin 5 mM/IG	DB ≈ 46 mg; PTC ≈ 15 mg; PTU, ≈8 mg; quinine ≈ 5 mg; Salicin ≈ 15 mg	Ad libitum food	↑ Food intake (first 30 min) ↓ Food intake (next 4 h)	[14]

CHO, carbohydrate; DB, denatonium benzoate; F, fat; HCl, hydrochloride; IG, intragastric; Prot, protein; PTC, phenylthiocarbamide; PTU, propylthiouracil.

## References

[1] Avau B., Bauters D., Steensels S., Vancleef L., Laermans J., Lesuisse J., Buyse J., Lijnen H.R., Tack J., Depoortere I. (2015). The Gustatory Signaling Pathway and Bitter Taste Receptors Affect the Development of Obesity and Adipocyte Metabolism in Mice. PLoS ONE.

[2] Chen M.C., Wu S.V., Reeve J.R., Rozengurt E. (2006). Bitter Stimuli Induce Ca^2+^ Signaling and CCK Release in Enteroendocrine STC-1 Cells: Role of L-Type Voltage-Sensitive Ca^2+^ Channels. Am. J. Physiology-Cell Physiol..

[3] Kim K.-S., Egan J.M., Jang H.-J. (2014). Denatonium Induces Secretion of Glucagon-Like Peptide-1 through Activation of Bitter Taste Receptor Pathways. Diabetologia.

[4] Marathe C.S., Rayner C.K., Jones K.L., Horowitz M. (2013). Relationships between Gastric Emptying, Postprandial Glycemia, and Incretin Hormones. Diabetes Care.

[5] Steinert R.E., Feinle-Bisset C., Asarian L., Horowitz M., Beglinger C., Geary N. (2017). Ghrelin, CCK, GLP-1, and PYY(3–36): Secretory Controls and Physiological Roles in Eating and Glycemia in Health, Obesity, and After RYGB. Physiol. Rev..

[6] Murphy K.G., Bloom S.R. (2006). Gut Hormones and the Regulation of Energy Homeostasis. Nat. Cell Biol..

[7] Seimon R.V., Lange K., Little T.J., Brennan I.M., Pilichiewicz A.N., Feltrin K.L., Smeets A.J., Horowitz M., Feinle-Bisset C. (2010). Pooled-Data Analysis Identifies Pyloric Pressures and Plasma Cholecystokinin Concentrations as Major Determinants of Acute Energy Intake in Healthy, Lean Men. Am. J. Clin. Nutr..

[8] Unick J.L., Beavers D., Bond D.S., Clark J.M., Jakicic J.M., Kitabchi A.E., Knowler W.C., Wadden T.A., Wagenknecht L.E., Wing R.R. (2013). The Long-Term Effectiveness of a Lifestyle Intervention in Severely Obese Individuals. Am. J. Med..

[9] Bhat S.P., Sharma A. (2017). Current Drug Targets in Obesity Pharmacotherapy-A Review. Curr. Drug Targets.

[10] Drucker D.J., Nauck M.A. (2006). The Incretin System: Glucagon-Like Peptide-1 Receptor Agonists and Dipeptidyl Peptidase-4 Inhibitors in Type 2 Diabetes. Lancet.

[11] Müller T., Finan B., Bloom S., D’Alessio D., Drucker D., Flatt P., Fritsche A., Gribble F., Grill H., Habener J. (2019). Glucagon-Like Peptide 1 (GLP-1). Mol. Metab..

[12] Deacon C.F., Mannucci E., Ahrén B. (2012). Glycaemic Efficacy of Glucagon-Like Peptide-1 Receptor Agonists and Dipeptidyl Peptidase-4 Inhibitors as Add-on Therapy to Metformin in Subjects with Type 2 Diabetes-a Review and Meta Analysis. Diabetes Obes. Metab..

[13] Le Nevé B., Foltz M., Daniel H., Gouka R. (2010). The Steroid Glycoside H.g.-12 from Hoodia Gordonii Activates the Human Bitter Receptor TAS2R14 and Induces CCK Release from HuTu-80 cells. Am. J. Physiol. Liver Physiol..

[14] Janssen S., Laermans J., Verhulst P.-J., Thijs T., Tack J., Depoortere I. (2011). Bitter Taste Receptors and α-Gustducin Regulate the Secretion of Ghrelin with Functional Effects on Food Intake and Gastric Emptying. Proc. Natl. Acad. Sci. USA.

[15] Avau B., Rotondo A., Thijs T., Andrews C.N., Janssen P., Tack J., Depoortere I. (2015). Targeting Extra-Oral Bitter Taste Receptors Modulates Gastrointestinal Motility with Effects on Satiation. Sci. Rep..

[16] Andreozzi P., Sarnelli G., Pesce M., Zito F.P., Alessandro A.D., Verlezza V., Palumbo I., Turco F., Esposito K., Cuomo R. (2015). The Bitter Taste Receptor Agonist Quinine Reduces Calorie Intake and Increases the Postprandial Release of Cholecystokinin in Healthy Subjects. J. Neurogastroenterol. Motil..

[17] Bitarafan V., Fitzgerald P.C.E., Little T.J., Meyerhof W., Jones K.L., Wu T., Horowitz M., Feinle-Bisset C. (2020). Intragastric Administration of the Bitter Tastant Quinine Lowers the Glycemic Response to a Nutrient Drink without Slowing Gastric Emptying in Healthy Men. Am. J. Physiol. Integr. Comp. Physiol..

[18] Iven J., Biesiekierski J.R., Zhao D., Deloose E., O’Daly O.G., Depoortere I., Tack J., Van Oudenhove L. (2018). Intragastric Quinine Administration decreases Hedonic Eating in Healthy Women through Peptide-Mediated Gut-Brain Signaling Mechanisms. Nutr. Neurosci..

[19] Deloose E., Janssen P., Corsetti M., Biesiekierski J., Masuy I., Rotondo A., Van Oudenhove L., Depoortere I., Tack J. (2017). Intragastric Infusion of Denatonium Benzoate Attenuates Interdigestive Gastric Motility and Hunger Scores in Healthy Female Volunteers. Am. J. Clin. Nutr..

[20] Rose B.D., Bitarafan V., Rezaie P., Fitzgerald P.C.E., Horowitz M., Feinle-Bisset C. (2021). Comparative Effects of Intragastric and Intraduodenal Administration of Quinine on the Plasma Glucose Response to a Mixed-Nutrient Drink in Healthy Men: Relations with Glucoregulatory Hormones and Gastric Emptying. J. Nutr..

[21] Bachmanov A.A., Beauchamp G.K. (2007). Taste Receptor Genes. Annu. Rev. Nutr..

[22] Depoortere I. (2014). Taste Receptors of the Gut: Emerging Roles in Health and Disease. Gut.

[23] Lu P., Zhang C.-H., Lifshitz L.M., Zhuge R. (2017). Extraoral Bitter Taste Receptors in Health and Disease. J. Gen. Physiol..

[24] Adler E., Hoon M.A., Mueller K.L., Chandrashekar J., Ryba N.J., Zuker C.S. (2000). A Novel Family of Mammalian Taste Receptors. Cell.

[25] Wu S.V., Rozengurt N., Yang M., Young S.H., Sinnett-Smith J., Rozengurt E. (2002). Expression of Bitter Taste Receptors of the T2R Family in the Gastrointestinal Tract and Enteroendocrine STC-1 cells. Proc. Natl. Acad. Sci. USA.

[26] Behrens M., Meyerhof W., Meyerhof W., Beisiegel U., Joost H.-G. (2010). Oral and Extraoral Bitter Taste Receptors.

[27] Drewnowski A., Gomez-Carneros C. (2000). Bitter Taste, Phytonutrients, and the Consumer: A Review. Am. J. Clin. Nutr..

[28] Maehashi K., Huang L. (2009). Bitter Peptides and Bitter Taste Receptors. Cell. Mol. Life Sci..

[29] Hofmann T. (2005). Taste-Active Maillard Reaction Products: The “Tasty” World of Nonvolatile Maillard Reaction Products. Ann. N. Y. Acad. Sci..

[30] Dubois G., DeSimone J., Lyall V. (2020). Chemistry of Gustatory Stimuli.

[31] Belitz H., Wieser H. (1985). Bitter Compounds: Occurrence and Structure-Activity Relationships. Food Rev. Int..

[32] Duffy V.B., Davidson A.C., Kidd J.R., Kidd K.K., Speed W.C., Pakstis A.J., Reed D.R., Snyder D.J., Bartoshuk L.M. (2004). Bitter Receptor Gene (TAS2R38), 6-n-Propylthiouracil (PROP) Bitterness and Alcohol Intake. Alcohol. Clin. Exp. Res..

[33] Keller K.L., Adise S. (2016). Variation in the Ability to Taste Bitter Thiourea Compounds: Implications for Food Acceptance, Dietary Intake, and Obesity Risk in Children. Annu. Rev. Nutr..

[34] Meyerhof W., Batram C., Kuhn C., Brockhoff A., Chudoba E., Bufe B., Appendino G., Behrens M. (2010). The Molecular Receptive Ranges of Human TAS2R Bitter Taste Receptors. Chem. Senses.

[35] Li F. (2013). Taste Perception: From the Tongue to the Testis. Mol. Hum. Reprod..

[36] Shi P., Zhang J. (2009). Extraordinary Diversity of Chemosensory Receptor Gene Repertoires among Vertebrates. Chemistry and Biology of Pteridines and Folates.

[37] Kim U., Wooding S., Ricci D., Jorde L.B., Drayna D. (2005). Worldwide Haplotype Diversity and Coding Sequence Variation at Human Bitter Taste Receptor Loci. Hum. Mutat..

[38] Bufe B., Breslin P.A., Kuhn C., Reed D.R., Tharp C.D., Slack J.P., Kim U.-K., Drayna D., Meyerhof W. (2005). The Molecular Basis of Individual Differences in Phenylthiocarbamide and Propylthiouracil Bitterness Perception. Curr. Biol..

[39] Kim U.-K., Jorgenson E., Coon H., Leppert M., Risch N., Drayna D. (2003). Positional Cloning of the Human Quantitative Trait Locus Underlying Taste Sensitivity to Phenylthiocarbamide. Science.

[40] Cvijanovic N., Feinle-Bisset C., Young R.L., Little T.J. (2015). Oral and Intestinal Sweet and Fat Tasting: Impact of Receptor Polymorphisms and Dietary Modulation for Metabolic Disease. Nutr. Rev..

[41] Hajishafiee M., Bitarafan V., Feinle-Bisset C. (2019). Gastrointestinal Sensing of Meal-Related Signals in Humans, and Dysregulations in Eating-Related Disorders. Nutrients.

[42] Latorre R., Sternini C., de Giorgio R., Meerveld B.G.-V. (2016). Enteroendocrine Cells: A Review of their Role in Brain-Gut Communication. Neurogastroenterol. Motil..

[43] Psichas A., Reimann F., Gribble F.M. (2015). Gut Chemosensing Mechanisms. J. Clin. Investig..

[44] Rasoamanana R., Darcel N., Fromentin G., Tomé D. (2012). Nutrient Sensing and Signalling by the Gut. Proc. Nutr. Soc..

[45] Vella A., Camilleri M. (2017). The Gastrointestinal Tract as an Integrator of Mechanical and Hormonal Response to Nutrient Ingestion. Diabetes.

[46] Symonds E.L., Peiris M., Page A.J., Chia B., Dogra H., Masding A., Galanakis V., Atiba M., Bulmer D., Young R.L. (2015). Mechanisms of Activation of Mouse and Human Enteroendocrine Cells by Nutrients. Gut.

[47] Gribble F.M., Reimann F. (2019). Function and Mechanisms of Enteroendocrine Cells and Gut Hormones in Metabolism. Nat. Rev. Endocrinol..

[48] Sternini C., Anselmi L., Rozengurt E. (2008). Enteroendocrine Cells: A Site of ‘Taste’ in Gastrointestinal Chemosensing. Curr. Opin. Endocrinol. Diabetes Obes..

[49] Imai H., Hakukawa M., Hayashi M., Iwatsuki K., Masuda K. (2020). Expression of Bitter Taste Receptors in the Intestinal Cells of Non-Human Primates. Int. J. Mol. Sci..

[50] Pham H., Hui H., Morvaridi S., Cai J., Zhang S., Tan J., Wu V., Levin N., Knudsen B., Goddard W.A. (2016). A Bitter Pill for Type 2 Diabetes? The Activation of Bitter Taste Receptor TAS2R38 can Stimulate GLP-1 Release from Enteroendocrine L-Cells. Biochem. Biophys. Res. Commun..

[51] Park J., Kim K.-S., Kim K.-H., Lee I.-S., Jeong H.-S., Kim Y., Jang H.-J. (2015). GLP-1 Secretion is Stimulated by 1,10-Phenanthroline via Colocalized T2R5 Signal Transduction in Human Enteroendocrine L Cell. Biochem. Biophys. Res. Commun..

[52] Wang Q., Liszt K.I., Deloose E., Canovai E., Thijs T., Farré R., Ceulemans L.J., Lannoo M., Tack J., Depoortere I. (2019). Obesity Alters Adrenergic and Chemosensory Signaling Pathways that Regulate Ghrelin Secretion in the Human Gut. FASEB J..

[53] Holst J.J., Gribble F., Horowitz M., Rayner C.K. (2016). Roles of the Gut in Glucose Homeostasis. Diabetes Care.

[54] Suzuki K., Jayasena C.N., Bloom S.R. (2011). The Gut Hormones in Appetite Regulation. J. Obes..

[55] Deloose E., Verbeure W., Depoortere I., Tack J. (2019). Motilin: From Gastric Motility Stimulation to Hunger Signalling. Nat. Rev. Endocrinol..

[56] Cummings D.E., Overduin J. (2007). Gastrointestinal Regulation of Food Intake. J. Clin. Investig..

[57] Cummings D.E. (2003). Roles for Ghrelin in the Regulation of Appetite and Body Weight. Arch. Surg..

[58] Field B.C.T., Chaudhri O.B., Bloom S.R. (2010). Bowels Control Brain: Gut Hormones and Obesity. Nat. Rev. Endocrinol..

[59] Abbott C.R., Small C.J., Kennedy A.R., Neary N.M., Sajedi A., Ghatei M.A., Bloom S.R. (2005). Blockade of the Neuropeptide Y Y2 Receptor with the Specific Antagonist BIIE0246 Attenuates the Effect of Endogenous and Exogenous Peptide YY(3–36) on Food Intake. Brain Res..

[60] Beglinger C., Degen L., Matzinger D., D’Amato M., Drewe J. (2001). Loxiglumide, a CCK-A Receptor Antagonist, Stimulates Calorie Intake and Hunger Feelings in Humans. Am. J. Physiol. Integr. Comp. Physiol..

[61] Steinert R.E., Schirra J., Meyer-Gerspach A.C., Kienle P., Fischer H., Schulte F., Goeke B., Beglinger C. (2014). Effect of Glucagon-Like Peptide-1 Receptor Antagonism on Appetite and Food Intake in Healthy Men. Am. J. Clin. Nutr..

[62] Degen L., Oesch S., Casanova M., Graf S., Ketterer S., Drewe J., Beglinger C. (2005). Effect of Peptide YY3–36 on Food Intake in Humans. Gastroenterology.

[63] Gutzwiller J.-P., Göke B., Drewe J., Hildebrand P., Ketterer S., Handschin D., Winterhalder R., Conen D., Beglinger C. (1999). Glucagon-Like Peptide-1: A Potent Regulator of Food Intake in Humans. Gut.

[64] MacIntosh C.G., Morley J.E., Wishart J., Morris H., Jansen J.B.M.J., Horowitz M., Chapman I.M. (2001). Effect of Exogenous Cholecystokinin (CCK)-8 on Food Intake and Plasma CCK, Leptin, and Insulin Concentrations in Older and Young Adults: Evidence for Increased CCK Activity as a Cause of the Anorexia of Aging. J. Clin. Endocrinol. Metab..

[65] De Lartigue G., Diepenbroek C. (2016). Novel Developments in Vagal Afferent Nutrient Sensing and its Role in Energy Homeostasis. Curr. Opin. Pharmacol..

[66] Dockray G.J. (2013). Enteroendocrine Cell Signalling via the Vagus Nerve. Curr. Opin. Pharmacol..

[67] Meloni A.R., Deyoung M.B., Lowe C., Parkes D.G. (2012). GLP-1 Receptor Activated Insulin Secretion from Pancreatic β-Cells: Mechanism and Glucose Dependence. Diabetes Obes. Metab..

[68] Holst J.J. (2007). The Physiology of Glucagon-like Peptide 1. Physiol. Rev..

[69] Kim W., Egan J.M. (2008). The Role of Incretins in Glucose Homeostasis and Diabetes Treatment. Pharmacol. Rev..

[70] Margolskee R.F. (2002). Molecular Mechanisms of Bitter and Sweet Taste Transduction. J. Biol. Chem..

[71] Xie C., Wang X., Young R.L., Horowitz M., Rayner C.K., Wu T. (2018). Role of Intestinal Bitter Sensing in Enteroendocrine Hormone Secretion and Metabolic Control. Front. Endocrinol..

[72] Ingram J.R., Walker E.G., Pahl M.C., Lo K.R., Shin H.S., Lang C., Wohlers M.W., Poppitt S., Sutton K.H. (2016). Activation of Gastrointestinal Bitter Taste Receptors Suppresses Food Intake and Stimulates Secretion of Gastrointestinal Peptide Hormones in Healthy Men. Obes. Facts.

[73] Mennella I., Fogliano V., Ferracane R., Arlorio M., Pattarino F., Vitaglione P. (2016). Microencapsulated Bitter Compounds (from Gentiana Lutea) Reduce Daily Energy Intakes in Humans. Br. J. Nutr..

[74] Van Avesaat M., Troost F.J., Ripken D., Peters J., Hendriks H.F., Masclee A.A. (2015). Intraduodenal Infusion of a Combination of Tastants Decreases Food Intake in Humans. Am. J. Clin. Nutr..

[75] Deloose E., Corsetti M., Van Oudenhove L., Depoortere I., Tack J. (2017). Intragastric Infusion of the Bitter Tastant Quinine Suppresses Hormone Release and Antral Motility during the Fasting State in Healthy Female Volunteers. Neurogastroenterol. Motil..

[76] Bitarafan V.E., Fitzgerald P.C., Little T.J., Meyerhof W., Wu T., Horowitz M., Feinle-Bisset C. (2019). Effects of Intraduodenal Infusion of the Bitter Tastant, Quinine, on Antropyloroduodenal Motility, Plasma Cholecystokinin, and Energy Intake in Healthy Men. J. Neurogastroenterol. Motil..

[77] Jeon T.-I., Seo Y.-K., Osborne T.F. (2011). Gut Bitter Taste Receptor Signalling induces ABCB1 through a Mechanism Involving CCK. Biochem. J..

[78] Yamazaki T., Morimoto-Kobayashi Y., Koizumi K., Takahashi C., Nakajima S., Kitao S., Taniguchi Y., Katayama M., Ogawa Y. (2019). Secretion of a Gastrointestinal Hormone, Cholecystokinin, by Hop-Derived Bitter Components Activates Sympathetic Nerves in Brown Adipose Tissue. J. Nutr. Biochem..

[79] Grau-Bové C., Miguéns-Gómez A., González-Quilen C., Fernández-López J.-A., Remesar X., Torres-Fuentes C., Ávila-Román J., Rodríguez-Gallego E., Beltrán-Debón R., Blay M.T. (2020). Modulation of Food Intake by Differential TAS2R Stimulation in Rat. Nutrients.

[80] Yue X., Liang J., Gu F., Du D., Chen F. (2018). Berberine Activates Bitter Taste Responses of Enteroendocrine STC-1 Cells. Mol. Cell. Biochem..

[81] Yu Y., Hao G., Zhang Q., Hua W., Wang M., Zhou W., Zong S., Huang M., Wen X. (2015). Berberine Induces GLP-1 Secretion through Activation of Bitter Taste Receptor Pathways. Biochem. Pharmacol..

[82] Serrano J., Casanova-Martí À., Depoortere I., Blay M.T., Terra X., Pinent M., Ardévol A. (2016). Subchronic Treatment with Grape-Seed Phenolics Inhibits Ghrelin Production despite a Short-Term Stimulation of Ghrelin Secretion Produced by Bitter-Sensing Flavanols. Mol. Nutr. Food Res..

[83] Suh H.-W., Lee K.-B., Kim K.-S., Yang H.J., Choi E.-K., Shin M.H., Park Y.S., Na Y.-C., Ahn K.S., Jang Y.P. (2015). A Bitter Herbal Medicine Gentiana Scabra Root Extract Stimulates Glucagon-Like Peptide-1 Secretion and Regulates Blood Glucose in db/db Mouse. J. Ethnopharmacol..

[84] Kok B.P., Galmozzi A., Littlejohn N.K., Albert V., Godio C., Kim W., Kim S.M., Bland J.S., Grayson N., Fang M. (2018). Intestinal Bitter Taste Receptor Activation Alters Hormone Secretion and Imparts Metabolic Benefits. Mol. Metab..

[85] Dotson C.D., Zhang L., Xu H., Shin Y.-K., Vigues S., Ott S.H., Elson A.E.T., Choi H.J., Shaw H., Egan J.M. (2008). Bitter Taste Receptors Influence Glucose Homeostasis. PLoS ONE.

[86] Li J., Xu J., Hou R., Jin X., Wang J., Yang N., Yang L., Liu L., Tao F., Lu H. (2017). Qing-Hua Granule induces GLP-1 Secretion via Bitter Taste Receptor in db/db Mice. Biomed. Pharmacother..

[87] Huang T.-N., Lu K.-N., Pai Y.-P., Hsu C., Huang C.-J. (2013). Role of GLP-1 in the Hypoglycemic Effects of Wild Bitter Gourd. Evidence-Based Complement. Altern. Med..

[88] Habib A.M., Richards P., Rogers G.J., Reimann F., Gribble F.M. (2013). Co-Localisation and Secretion of Glucagon-Like Peptide 1 and Peptide YY from Primary Cultured Human L Cells. Diabetologia.

[89] Lossow K., Hübner S., Roudnitzky N., Slack J.P., Pollastro F., Behrens M., Meyerhof W. (2016). Comprehensive Analysis of Mouse Bitter Taste Receptors Reveals Different Molecular Receptive Ranges for Orthologous Receptors in Mice and Humans. J. Biol. Chem..

[90] Azpiroz F., Malagelada J.R. (1985). Intestinal Control of Gastric Tone. Am. J. Physiol. Liver Physiol..

[91] Houghton L., Read N., Heddle R., Horowitz M., Collins P., Chatterton B., Dent J. (1988). Relationship of the Motor Activity of the Antrum, Pylorus, and Duodenum to Gastric Emptying of a Solid-Liquid Mixed Meal. Gastroenterology.

[92] Brookes S.J., Spencer N.J., Costa M., Zagorodnyuk V.P. (2013). Extrinsic Primary Afferent Signalling in the Gut. Nat. Rev. Gastroenterol. Hepatol..

[93] Feinle C., Grundy D., Read N.W. (1997). Effects of Duodenal Nutrients on Sensory and Motor Responses of the Human Stomach to Distension. Am. J. Physiol. Content.

[94] Kissileff H.R., Carretta J.C., Geliebter A., Pi-Sunyer F.X. (2003). Cholecystokinin and Stomach Distension Combine to Reduce Food Intake in Humans. Am. J. Physiol. Integr. Comp. Physiol..

[95] Sturm K., Parker B., Wishart J., Feinle-Bisset C., Jones K.L., Chapman I., Horowitz M. (2004). Energy Intake and Appetite are Related to Antral Area in Healthy Young and Older Subjects. Am. J. Clin. Nutr..

[96] Horowitz M., Edelbroek M.A.L., Wishart J.M., Straathof J.W. (1993). Relationship between Oral Glucose Tolerance and Gastric Emptying in Normal Healthy Subjects. Diabetologia.

[97] Jones K.L., Horowitz M.I., Carney B., Wishart J.M., Guha S., Green L. (1996). Gastric Emptying in Early Noninsulin-Dependent Diabetes Mellitus. J. Nucl. Med..

[98] Little T.J., Pilichiewicz A.N., Russo A., Phillips L., Jones K.L., Nauck M.A., Wishart J., Horowitz M., Feinle-Bisset C. (2006). Effects of Intravenous Glucagon-Like Peptide-1 on Gastric Emptying and Intragastric Distribution in Healthy Subjects: Relationships with Postprandial Glycemic and Insulinemic Responses. J. Clin. Endocrinol. Metab..

[99] Nauck M.A., Niedereichholz U., Ettler R., Holst J.J., Ørskov C., Ritzel R., Schmiegel W.H. (1997). Glucagon-Like Peptide 1 Inhibition of Gastric Emptying Outweighs its Insulinotropic Effects in Healthy Humans. Am. J. Physiol. Metab..

[100] Kimura Y., Sumiyoshi M. (2011). Effects of Swertia Japonica Extract and its Main Compound Swertiamarin on Gastric Emptying and Gastrointestinal Motility in Mice. Fitoterapia.

[101] Harada Y., Koseki J., Sekine H., Fujitsuka N., Kobayashi H. (2019). Role of Bitter Taste Receptors in Regulating Gastric Accommodation in Guinea Pigs. J. Pharmacol. Exp. Ther..

[102] Glendinning J.I., Yiin Y.-M., Ackroff K., Sclafani A. (2008). Intragastric Infusion of Denatonium Conditions Flavor Aversions and Delays Gastric Emptying in Rodents. Physiol. Behav..

[103] Little T.J., Gupta N., Case R.M., Thompson D.G., McLaughlin J.T. (2009). Sweetness and Bitterness Taste of Meals Per se does not Mediate Gastric Emptying in Humans. Am. J. Physiol. Integr. Comp. Physiol..

[104] Wicks D., Wright J., Rayment P., Spiller R. (2005). Impact of Bitter Taste on Gastric Motility. Eur. J. Gastroenterol. Hepatol..

[105] Sumiyoshi M., Kimura Y. (2013). Hop (Humulus Lupulus L.) Extract Inhibits Obesity in Mice Fed a High-Fat Diet over the Long Term. Br. J. Nutr..

[106] Yajima H., Noguchi T., Ikeshima E., Shiraki M., Kanaya T., Tsuboyama-Kasaoka N., Ezaki O., Oikawa S., Kondo K. (2005). Prevention of Diet-Induced Obesity by Dietary Isomerized Hop Extract Containing Isohumulones, in Rodents. Int. J. Obes..

[107] Van Heerden F.R., Horak R.M., Maharaj V.J., Vleggaar R., Senabe J.V., Gunning P.J. (2007). An Appetite Suppressant from Hoodia Species. Phytochemistry.

[108] Leng S.-H., Lu F.-E., Xu L.-J. (2004). Therapeutic Effects of Berberine in Impaired Glucose Tolerance Rats and its Influence on Insulin Secretion. Acta Pharmacol. Sin..

[109] Kratz C.M., Levitsky D., Lustick S.L. (1978). Long Term Effects of Quinine on Food Intake and Body Weight in the Rat. Physiol. Behav..

[110] Yajima H., Ikeshima E., Shiraki M., Kanaya T., Fujiwara D., Odai H., Tsuboyama-Kasaoka N., Ezaki O., Oikawa S., Kondo K. (2004). Isohumulones, Bitter Acids Derived from Hops, Activate Both Peroxisome Proliferator-Activated Receptor α and γ and Reduce Insulin Resistance. J. Biol. Chem..

[111] Nauck M.A., Kleine N., Holst J.J., Willms B., Creutzfeldt W. (1993). Normalization of Fasting Hyperglycaemia by Exogenous Glucagon-Like Peptide 1 (7-36 Amide) in Type 2 (Non-Insulin-Dependent) Diabetic Patients. Diabetologia.

[112] Henquin J., Horemans B., Nenquin M., Verniers J., Lambert A. (1975). Quinine-Induced Modifications of Insulin Release and Glucose Metabolism by Isolated Pancreatic Islets. FEBS Lett..

[113] Vignini A., Borroni F., Sabbatinelli J., Pugnaloni S., Alia S., Taus M., Ferrante L., Mazzanti L., Fabri M. (2019). General Decrease of Taste Sensitivity Is Related to Increase of BMI: A Simple Method to Monitor Eating Behavior. Dis. Markers.

[114] Bianchi L.L., Galmarini M., García-Burgos D., Zamora M. (2018). Time-Intensity and Reaction-Time Methodology Applied to the Dynamic Perception and Liking of Bitterness in Relation to Body Mass Index. Food Res. Int..

[115] Garcia-Burgos D., Zamora M. (2013). Facial Affective Reactions to Bitter-Tasting Foods and Body Mass Index in Adults. Appetite.

[116] Simchen U., Koebnick C., Hoyer S., Issanchou S., Zunft H.-J.F. (2006). Odour and Taste Sensitivity is Associated with Body Weight and Extent of Misreporting of Body Weight. Eur. J. Clin. Nutr..

[117] Overberg J., Hummel T., Krude H., Wiegand S. (2012). Differences in Taste Sensitivity between Obese and Non-obese Children and Adolescents. Arch. Dis. Child..

[118] De Carli L., Gambino R., Lubrano C., Rosato R., Bongiovanni D., Lanfranco F., Broglio F., Ghigo E., Bo S. (2017). Impaired Taste Sensation in Type 2 Diabetic Patients without Chronic Complications: A Case–Control Study. J. Endocrinol. Investig..

[119] Matsugasumi M., Hashimoto Y., Okada H., Tanaka M., Kimura T., Kitagawa N., Tanaka Y., Fukuda Y., Sakai R., Yamazaki M. (2018). The Association between Taste Impairment and Serum Zinc Concentration in Adult Patients with Type 2 Diabetes. Can. J. Diabetes.

[120] Pugnaloni S., Alia S., Mancini M., Santoro V., Di Paolo A., Rabini R.A., Fiorini R., Sabbatinelli J., Fabri M., Mazzanti L. (2020). A Study on the Relationship between Type 2 Diabetes and Taste Function in Patients with Good Glycemic Control. Nutrients.

[121] Gondivkar S.M., Indurkar A., Degwekar S., Bhowate R. (2009). Evaluation of Gustatory Function in Patients with Diabetes Mellitus Type 2. Oral Surg. Oral Med. Oral Pathol. Oral Radiol. Endodontol..

[122] Wölnerhanssen B.K., Moran A.W., Burdyga G., Meyer-Gerspach A.C., Peterli R., Manz M., Thumshirn M., Daly K., Beglinger C., Shirazi-Beechey S.P. (2017). Deregulation of Transcription Factors Controlling Intestinal Epithelial Cell Differentiation; a Predisposing Factor for Reduced Enteroendocrine Cell Number in Morbidly Obese Individuals. Sci. Rep..

[123] Latorre R., Huynh J., Mazzoni M., Gupta A., Bonora E., Clavenzani P., Chang L., Mayer E.A., de Giorgio R., Sternini C. (2016). Expression of the Bitter Taste Receptor, T2R38, in Enteroendocrine Cells of the Colonic Mucosa of Overweight/Obese vs. Lean Subjects. PLoS ONE.

[124] Keller M., Liu X., Wohland T., Rohde K., Gast M.-T., Stumvoll M., Kovacs P., Tönjes A., Böttcher Y. (2013). TAS2R38 and Its Influence on Smoking Behavior and Glucose Homeostasis in the German Sorbs. PLoS ONE.

[125] Spector A.C., Kopka S.L. (2002). Rats Fail to Discriminate Quinine from Denatonium: Implications for the Neural Coding of Bitter-Tasting Compounds. J. Neurosci..

